# First report of severe acute respiratory syndrome coronavirus 2 detection in two asymptomatic cats in the state of Pernambuco, Northeastern Brazil

**DOI:** 10.14202/vetworld.2021.2839-2842

**Published:** 2021-10-31

**Authors:** Ivyson da Silva Epifanio, Davi dos Santos Rodrigues, Leonardo Borges de Lima, Maria Aurea de Azevedo Nogueira, Laelia Reginae do Monte Pessoa Felix, Barbara Ferreira de Almeida, Claudia Kathariny da Silva Farias, Otavio Valerio de Carvalho, Rita de Cassia Carvalho Maia, Luiz Eduardo Ristow, David Soeiro Barbosa, Juliana Arena Galhardo, Christina Pettan-Brewer, Louise Bach Kmetiuk, Rafael Garabet Agopian, Valeria Dutra, Helio Autran de Morais, Andrea Pires dos Santos, Alexander Welker Biondo, Daniel Friguglietti Brandespim

**Affiliations:** 1 Department of Veterinary Medicine, College of Veterinary Medicine, Federal Rural University of Pernambuco, Recife, Pernambuco, Brazil; 2 TECSA Animal Reference Laboratory, Belo Horizonte, Minas Gerais, Brazil; 3 Department of Parasitology, Institute of Biological Sciences, Federal University of Minas Gerais, Belo Horizonte, Minas Gerais, Brazil; 4 Department of Veterinary Medicine, College of Veterinary Medicine, Federal University of Mato Grosso do Sul, Campo Grande, Mato Grosso do Sul, Brazil; 5 Department of Comparative Medicine, School of Medicine, University of Washington, Seattle, Washington, USA; 6 Department of Veterinary Medicine, Federal University of Paran, Curitiba, Paran, Brazil; 7 Department of Veterinary Medicine, University of Santo Amaro, São Paulo, Brazil; 8 Department of Veterinary Medicine, College of Veterinary Medicine, Federal University of Mato Grosso, Cuiab, Mato Grosso, Brazil; 9 Department of Clinical Sciences, Oregon State University, Corvallis, Oregon, USA; 10 Department of Comparative Pathobiology, College of Veterinary Medicine, Purdue University, West Lafayette, Indiana, USA.

**Keywords:** Brazil, coronavirus, domestic animals, pandemic

## Abstract

**Background and Aim:**

Despite worldwide case reports, including Brazilian cases, no frequency study on infection of pets by severe acute respiratory syndrome coronavirus 2 (SARS-CoV-2) has been conducted to date in Brazil. Accordingly, the present study was aimed to assess dogs and cats belonging to positive owners in Recife, Northeastern Brazil.

**Materials and Methods:**

This was a longitudinal prospective study on dogs and cats in the city of Recife whose owners were in isolation at home due to a confirmed laboratory diagnosis of SARS-CoV-2 through reverse-transcriptase polymerase chain reaction (RT-qPCR). Oral and rectal swabs from the pets were tested for the presence of SARS-CoV-2-specific RNA by means of RT-qPCR.

**Results:**

Among the pets tested, 0/16 dogs and 2/15 cats were positive for SARS-CoV-2. Interestingly, the two positive cats were owned by two unrelated asymptomatic veterinary students, which, therefore, post a warning to veterinarians worldwide.

**Conclusion:**

The findings herein indicate that cats may act as sentinels for human cases, particularly sharing households with asymptomatic human cases. Although with small sampling and convenient recruiting, the presence of infected cats by SARS-CoV-2 was most likely due to close cat-human contact with positive owners, posting a human-animal health threat when pets share the same bed and interact with owners without protection, particularly during owner self-isolation. Thus, infected owners should follow the same human preventive guidelines with their pets to avoid spreading infection.

## Introduction

The novel coronavirus (severe acute respiratory syndrome coronavirus or severe acute respiratory syndrome coronavirus 2 [SARS-CoV-2]), which has been recognized as an animal-borne pathogen, was first detected in humans in China in December 2019 [1]. In March 2020, the first cat with SARS-CoV-2 infection, which was clinically healthy and screened only due to its owner’s hospitalization, was reported in Hong Kong [2]. Since then, 115 cases have been identified in cats with or without clinical signs, and these cases have mostly arisen in cats living in close contact with infected humans [2]. Although death has reportedly been rare, it has been confirmed that two cats died due to SARS-CoV-2 infection: A 4-month-old female Ragdoll kitten (out of 387 cats tested) belonging to a clinically ill but untested owner in March 2020 [3], and one cat (out of a total of 3,625 animals tested, among which 94 were positive) with no pre-existing conditions, which was subsequently euthanized due to several clinical conditions. Regarding this last case, it was concluded that, in rare circumstances, SARS-CoV-2 might contribute to or cause death among cats with comorbid conditions [4]. Nonetheless, transmission from cats to human beings has not been reported anywhere around the world to date.

In cats, SARS-CoV-2 penetrates cells by binding a viral spike protein, which has high homology between cat and human receptors, to the angiotensin-converting enzyme 2 receptor [5]. Experimentally inoculated cats have not shown any clinical signs but have shown viral RNA shedding of SARS-CoV-2 in nasopharyngeal and fecal secretions, along with immunoglobulin G antibodies, 24 days after intranasal inoculation [6]. These inoculated cats have also successfully infected cohoused cats, causing severe tracheal and pulmonary lesions in susceptible youngsters [6].

Despite these cases described worldwide, the role of cats in SARS-CoV-2 infection remains to be fully established. Accordingly, as part of an ongoing project to evaluate the impact of SARS-CoV-2 in pets exposed to infected owners in Brazil, the aim of this study was to describe SARS-CoV-2 infection in two asymptomatic cats in Recife, the capital of the state of Pernambuco, in Northeastern Brazil.

## Materials and Methods

### Ethical approval

The present study was approved by the Ethics Committee for Animal Use (protocol number 4879280420) and the Ethics Committee for Research on Human Beings (protocol number 4.054.208) of the Federal Rural University of Pernambuco.

### Study period and location

The study was conducted in December 2020. This was a longitudinal prospective study on dogs and cats conducted in the city of Recife, whose owners were in isolation at home due to a confirmed laboratory diagnosis of SARS-CoV-2 through reverse-transcriptase polymerase chain reaction (RT-qPCR). Recife (08°04’03” S; 34°55’00” W) is an Atlantic coastal city that is the capital and largest city of the state of Pernambuco, and it is currently the fifth largest urban area in Brazil. The city is ranked 9^th^ in population with 4,054,866 inhabitants; 210^th^ in human development index, with 0.772; and 13^th^ in gross domestic product, out of 5568 cities in Brazil. Recife has a tropical monsoon climate (Am) bordering on a tropical wet and dry climate (As) under the Köppen climate classification, with average temperatures of 22°C-30°C in the summer and 21°C-27°C in the winter, and high relative humidity throughout the year.

### Sampling

Visits were made to these households after receiving voluntary agreement from the owners. Samples were taken from their pets by means of oral, nasal, and rectal swabs, placed in transportation media and immediately sent for RT-qPCR testing.

The swab samples were evaluated for the presence of SARS-CoV-2-specific RNA at the TECSA Laboratories (Belo Horizonte, Brazil). Briefly, collection tubes containing swab samples and viral transportation medium were vortexed for 30 s, and 500 μL were used for RNA isolation using a magnetic bead-based nucleic acid extraction kit. Total RNA from the swab samples was extracted using a commercially available kit (Maxwell^®^ RSC simplyRNA Tissue Kit, Promega Co., Madison, WI, USA) and an automated platform (Maxwell^®^ RSC 48, Promega Co., Madison, WI, USA), in accordance with the manufacturer’s instructions. SARS-CoV-2 RT-qPCR tests were performed using a commercially available (GoTaq^®^ 1-Step RT- Promega Co., Madison, WI, USA) and a commercial kit (2019-nCoV, Integrated DNA Technologies – IDT, Coralville, IA, USA) targeting two regions: 2019 nCoV_N1 (F: GACCCCAAAATCAGCGAAAT, R: TCTGGTTACTGCCAGTTGAATCTG and P: ACCCCGCATTACGTTTGGTGGACC) and 2019 nCoV_N2 (F: TTACAAACATTGGCCGCAAA, R: GCGCGACATTCCGAAGAA and P: ACAATTTGCCCCCAGCGCTTCAG) as previously described for the detection of SARS-CoV-2 [7]. RT-qPCR was carried out in a commercial thermocycler (QuantStudio™ 1 – 96-Well 0.2 mL Block, Thermo Fisher, Waltham, MA, USA). The cycling consisted of a holding stage of 50°C for 15 min and 95°C for 2 min, followed by 45 cycles of 95°C for 3 s and 55°C for 30 s. A cycle threshold value (CT-value) of less than 40 was defined as a positive test result. All samples were tested in duplicate and were only considered positive for SARS-CoV-2 when at least two genes were amplified. Samples with only one target amplified were considered inconclusive. Quantification of SARS-CoV-2 RNA was performed using the same reaction. A commercial control (2019-nCoV Positive Control, Integrated DNA Technologies – IDT, Coralville, IA, USA) was used to provide a standard curve (5-dilution points), and the feline β-actin (ACTB) gene was used as an internal control gene.

## Results

Among the pets from positive owners that were tested, 0/16 dogs and 2/15 cats (RE-02F and RE-04F) tested positive for SARS-CoV-2 RT-qPCR. Both of these cats had positive oropharyngeal swabs, but RE-04F also had one positive target from the rectal swab (target N2: CT 36.7, 2.41 RNA copy number/μL). The oropharyngeal swab from RE-04F had a higher viral load (target N1: CT 31.65, 11.362 RNA copy number/μL; target N2: CT 33.46, 19.2 RNA copy number/μL) than the oropharyngeal swab from RE-02F (target N1: CT 34.49, 1.87 RNA copy number/μL; target N2: CT 34.38, 10.65 RNA copy number/μL). ACTB-specific amplification was successfully implemented in all assays.

Interestingly, these two cats were owned by two unrelated asymptomatic veterinary students, with close animal-human contact and cats sleeping on the same bed. Neither of these animals had any related clinical signs at the time of sampling. RE-02F was a 1-year-old female mixed-breed cat, and RE-04F was a 1-year-old male mixed-breed cat. No viral genome was successfully sequenced due to CT ≥30.0.

## Discussion

To the best of our knowledge, this is the first report showing cats positive for SARS-CoV-2 infection, detected by means of oropharyngeal and rectal swab samples in Northeastern Brazil [8]. The owned cats described here most likely became infected with SARS-CoV-2 due to close contact with infected owners, including through sharing the same bed and interacting without protection during self-isolation. Despite several attempts, no viral genome or sub-genomic fragments were successfully sequenced, probably due to low viral load of all cat samples, which varied from Ct 36.7, 34.49, 34.38, and 33.46 to 31.65, still higher than recommended by most commercial protocols of Ct 30.0 or lower. Unsuccessful sequencing herein has corroborated to human clinical samples in a recent study using a newly capture sequencing methodology to generate SARS-CoV-2 genomic and transcriptome sequences, of which complete genomes were generated only from samples with higher viral loads, with CT-value under 33, based on the CDC qPCR assay [9].

In a previous study in France, all nine cats and 12 dogs owned by 18 veterinary students tested negative for SARS-CoV-2 RNA and neutralizing antibodies, including 2/18 diagnosed with SARS-CoV-2 infection [10]. In a recent study in southeastern Brazil, all 49 cats (40 owned and nine stray) and 47 dogs (42 owned and 5 stray) were found to be negative for SARS-CoV-2 infection, but one stray cat and one dog presented neutralizing antibodies for the new coronavirus [11]. In another study, also in southeastern Brazil, 2/10 cats and 6/29 dogs were considered positive for molecular presence of SARS-CoV-2 [12].

Although the sample for this study was small and recruited according to convenience, the presence of infected cats demonstrated here corroborates the findings from other SARS-CoV-2 studies conducted worldwide, which have found that cats show higher susceptibility to infection than dogs [13,14]. Moreover, in those other studies, SARS-CoV-2 was also molecularly detected from rectal swab samples. Taken together, such findings may indicate that cats can act as sentinel animals for human cases of this disease, particularly with regard to households with asymptomatic human cases. Moreover, companion animals have so far not been implicated in human transmission, which may indicate that there is a lower risk of spillover back to humans [15].

The two positive cats were asymptomatic according to the sampling. They were unrelated, restricted to indoor life only, and lived as single cats in their respective households. Both cats were owned by veterinarians, who had the habits of hugging and kissing their cats, and sharing the same bed with them. Even after receiving their diagnoses of SARS-CoV-2, these owners continued to have regular close contact with their cats. Such contact with one or more SARS-CoV-2 infected persons have been reported to increase the risk of pet infection [16].

## Conclusion

This study provides the first report of SARS-CoV-2 infected cats, detected through oropharyngeal and rectal swab samples in Northeastern Brazil. Although this sample was small and recruited according to convenience sampling, the presence of cats infected by SARS-CoV-2 was most likely due to close cat-human contact with positive owners, including sharing the same bed and interacting without protection during self-isolation. Infected people should take the same precautions with their pets to avoid spreading infection during self-isolation.

## Authors’ Contributions

ISE, RCCM, CPB, LBK, RGA, VD, HAM, APS, AWB, and DFB: Conceptualization, conducted the study, and analyzed the samples. ISE, DSR, LBL, MAAN, LRMPF, BFA, CKSF, OVC, RCCM, LER, DSB, JAG, OVC, LER and DFB: Data collection and data analysis. All authors: writing of the original draft. All authors read, revised and approved the final manuscript.
